# Supplementary material for: Protocol for a realist review of General Practitioners’ Role in Advancing Practice in Care Homes (GRAPE study)
Source: BMJ Open. 2020 Jun 15;10(6):e036221. doi: 10.1136/bmjopen-2019-036221 (PMC7299033; doi:10.1136/bmjopen-2019-036221)
Supplement: Supplementary data [file bmjopen-2019-036221supp001.pdf]

Appendix

Initial search strategy for Medline

```
Database: Ovid MEDLINE(R) and Epub Ahead of Print, In-Process & Other
Non-Indexed Citations and Daily <1946 to October 18, 2019>
Search Strategy:
--------------------------------------------------------------------------------
1     Nursing home/ (8490)
2     nursing home*.tw. (28672)
3     homes for the aged/ (13479)
4     care home*.tw. (3640)
5     or/1-4 [care homes] (47826)
6     Long-Term Care/ (25324)
7     Residential facilities/ (5332)
8     ((long-term or longterm or retir*) adj5 (facility or facilities
or institution* or resident*)).tw. (10445)
9     or/6-8 [long term care or residential facility terms] (36831)
10     exp aged/ (3005375)
11     Geriatrics/ (29509)
12     Geriatric Nursing/ (13480)
13     health services for the aged/ (17340)
14     ((older or elder*) adj (person or people or adult* or patient*
or inpatient* or resident* or men or women)).tw. (237237)
15     (geriatric* or elderly or seniors or senior citizen* or
pensioner* or oldest old or very old).tw. (275140)
16     or/10-15 [old people] (3140511)
17     9 and 16 [long term or residential facilities for older people]
(17408)
18     5 or 17 [all care home or long term care or residential
facilities for older people] (60893)
19     (general adj (practice* or medical practitioner* or
practitioner*)).ti. (33662)
20     (general adj (practice* or medical practitioner* or
practitioner*)).ab. /freq=2 (18266)
21     (family adj (medicine or practice* or doctor* or physician* or
practitioner*)).ti. (14762)
22     (family adj (medicine or practice* or doctor* or physician* or
practitioner*)).ab. /freq=2 (9570)
23     Family Practice/ (64754)
24     physicians, Family/ (16156)
25     Physicians, Primary Care/ (3134)
26     GP.ti. (4505)
27     GP.ab. /freq=2 (22927)
28     (elderly adj2 physician*).ti. (52)
29     (elderly adj2 physician*).ab. /freq=2 (20)
30     Physician Executives/ (4198)
31     medical director*.ti. (706)
32     medical director*.ab. /freq=2 (409)
33     (primary adj1 care doctor*).ti. (195)
34     (primary adj1 care doctor*).ab. /freq=2 (143)
35     or/19-24 [gps or care home doctors] (102790)
36     exp Great Britain/ (357312)
37     (national health service* or nhs*).ti,ab,in. (179827)
38     (english not ((published or publication* or translat* or written
or language* or speak* or literature or citation*) adj5
english)).ti,ab. (92722)
```

39 (gb or "g.b." or britain* or (british* not "british columbia") or uk or "u.k." or united kingdom* or (england* not "new england") or northern ireland* or northern irish* or scotland* or scottish* or ((wales or "south wales") not "new south wales") or welsh*).ti,ab,jw,in. (1973824)

40 (bath or "bath's" or ((birmingham not alabama*) or ("birmingham's" not alabama*) or bradford or "bradford's" or brighton or "brighton's" or bristol or "bristol's" or carlisle* or "carlisle's" or (cambridge not (massachusetts* or boston* or harvard*)) or ("cambridge's" not (massachusetts* or boston* or harvard*)) or (canterbury not zealand*) or ("canterbury's" not zealand*) or chelmsford or "chelmsford's" or chester or "chester's" or chichester or "chichester's" or coventry or "coventry's" or derby or "derby's" or (durham not (carolina* or nc)) or ("durham's" not (carolina* or nc)) or ely or "ely's" or exeter or "exeter's" or gloucester or "gloucester's" or hereford or "hereford's" or hull or "hull's" or lancaster or "lancaster's" or leeds* or leicester or "leicester's" or (lincoln not nebraska*) or ("lincoln's" not nebraska*) or (liverpool not (new south wales* or nsw)) or ("liverpool's" not (new south wales* or nsw)) or ((london not (ontario* or ont or toronto*)) or ("london's" not (ontario* or ont or toronto*)) or manchester or "manchester's" or (newcastle not (new south wales* or nsw)) or ("newcastle's" not (new south wales* or nsw)) or norwich or "norwich's" or nottingham or "nottingham's" or oxford or "oxford's" or peterborough or "peterborough's" or plymouth or "plymouth's" or portsmouth or "portsmouth's" or preston or "preston's" or ripon or "ripon's" or salford or "salford's" or salisbury or "salisbury's" or sheffield or "sheffield's" or southampton or "southampton's" or st albans or stoke or "stoke's" or sunderland or "sunderland's" or truro or "truro's" or wakefield or "wakefield's" or wells or westminster or "westminster's" or winchester or "winchester's" or wolverhampton or "wolverhampton's" or (worcester not (massachusetts* or boston* or harvard*)) or ("worcester's" not (massachusetts* or boston* or harvard*)) or (york not ("new york*" or ny or ontario* or ont or toronto*)) or ("york's" not ("new york*" or ny or ontario* or ont or toronto*))))).ti,ab,in. (1327221)

41 (bangor or "bangor's" or cardiff or "cardiff's" or newport or "newport's" or st asaph or "st asaph's" or st davids or swansea or "swansea's").ti,ab,in. (51805)

42 (aberdeen or "aberdeen's" or dundee or "dundee's" or edinburgh or "edinburgh's" or glasgow or "glasgow's" or inverness or (perth not australia*) or ("perth's" not australia*) or stirling or "stirling's").ti,ab,in. (197789)

43 (armagh or "armagh's" or belfast or "belfast's" or lisburn or "lisburn's" or londonderry or "londonderry's" or derry or "derry's" or newry or "newry's").ti,ab,in. (24417)

44 or/36-43 (2542613)

45 (exp africa/ or exp americas/ or exp antarctic regions/ or exp arctic regions/ or exp asia/ or exp australia/ or exp oceania/) not (exp great britain/ or europe/) (2763442)

46 44 not 45 [UK publications search filter] (2403187)

47 18 and 35 and 46 (201)
